# Incidental Finding of an Extensive Type B Aortic Dissection Extending to the Iliac Arteries

**DOI:** 10.7759/cureus.22655

**Published:** 2022-02-27

**Authors:** Eric Landa, Saad Javaid, Frederick Campos, Erika Vigandt, Murtaza Hussaini

**Affiliations:** 1 Internal Medicine, Unity Health, Searcy, USA; 2 Internal Medicine, Wyckoff Heights Medical Center, Brooklyn, USA; 3 Internal Medicine, Ross University School of Medicine, Bridgetown, BRB

**Keywords:** internal medicine, cardiology, type b aortic dissection, dissection, aortic dissection

## Abstract

An aortic dissection is a life-threatening event that requires urgent evaluation. A dissection is defined as a tear in the innermost layer of the aortic wall forming a true and false lumen. This is normally diagnosed with a CT with contrast when clinical suspicion is present. Deciding whether urgent surgical intervention is required is key, as it may determine the survival of the patient. The treatment of type A aortic dissection involves emergent open-heart surgery. Medical treatment and clinical follow-up are recommended for uncomplicated type B dissections. In this report, we present a case of an extensive type B aortic dissection in an asymptomatic patient who required immediate surgical intervention.

## Introduction

Aortic dissections can be divided into types A and B based on the Stanford classification. Type A involves the ascending aorta and may progress to involve the arch and thoracoabdominal aorta while type B involves the descending thoracic or thoracoabdominal aorta distal to the left subclavian artery without the involvement of ascending aorta [1]. Individuals can present in the acute setting with a triad of symptoms typically including discrepancies in blood pressure between the two arms, sharp tearing pain, and mediastinal widening seen on chest X-ray. We present an interesting case of an incidental finding of an extensive type B aortic dissection extending from the thoracoabdominal aorta to the femoral arteries, which was found on a CT for the evaluation of a ventral hernia.

## Case presentation

**Figure 1:**
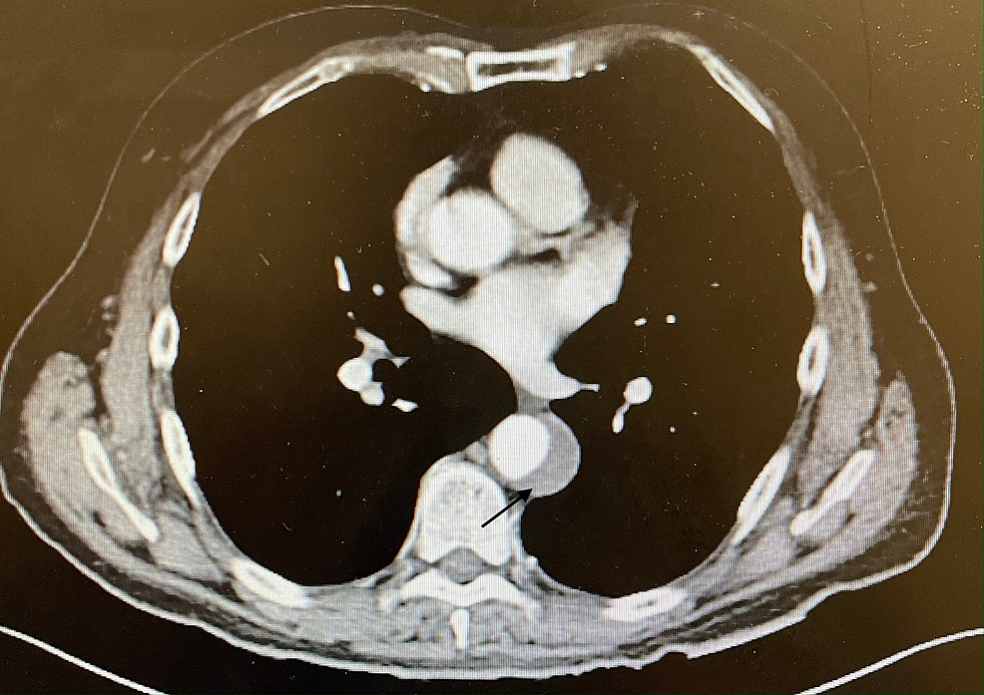
Focal arterial dissection along with thrombus extending upward in the descending thoracic aorta approaching the distal arch as seen on CT angiogram CT: computed tomography

**Figure 2:**
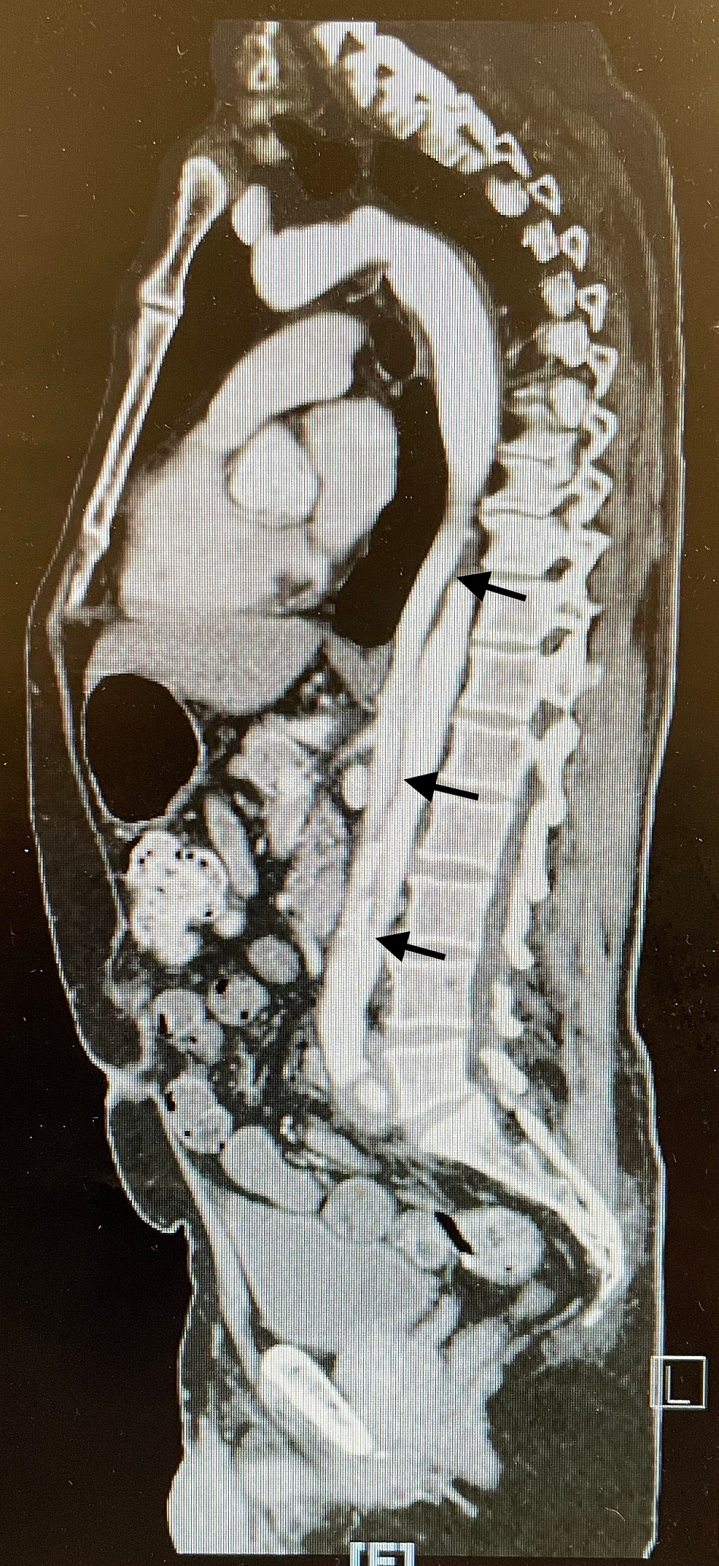
Aortic dissection as seen on CT angiogram CT: computed tomography

**Figure 3:**
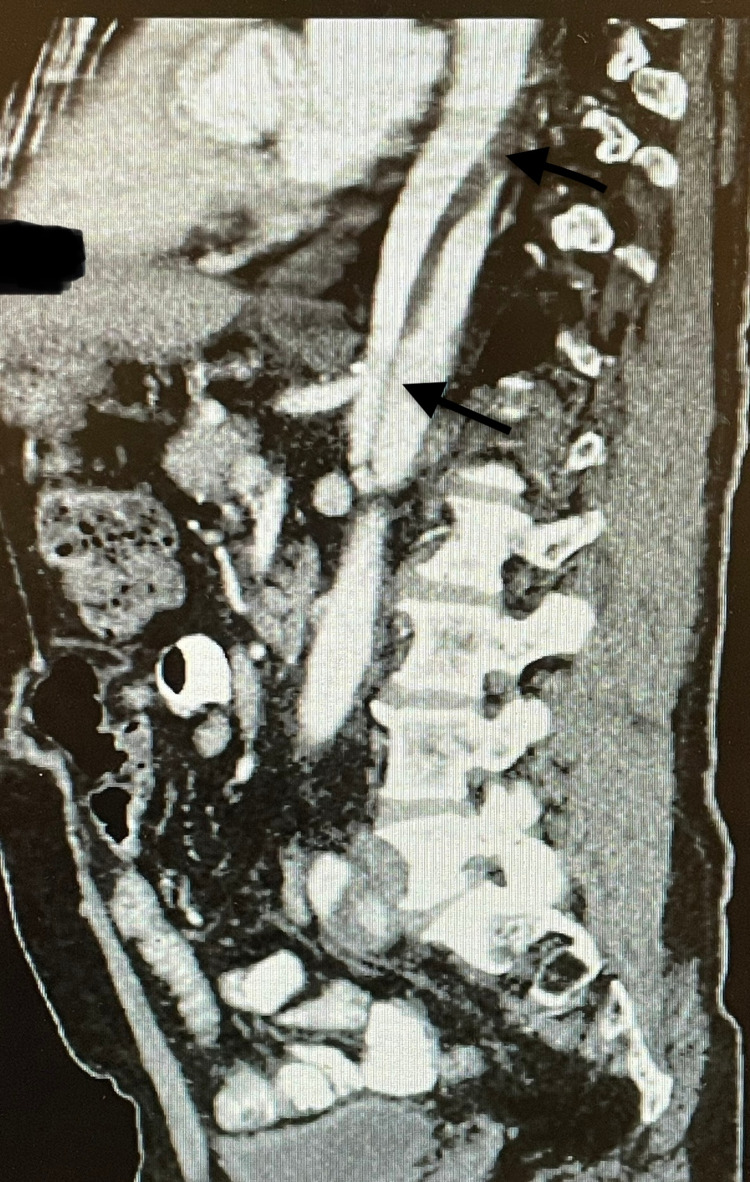
Extensive aortic dissection as seen on CT angiogram CT: computed tomography

**Figure 4:**
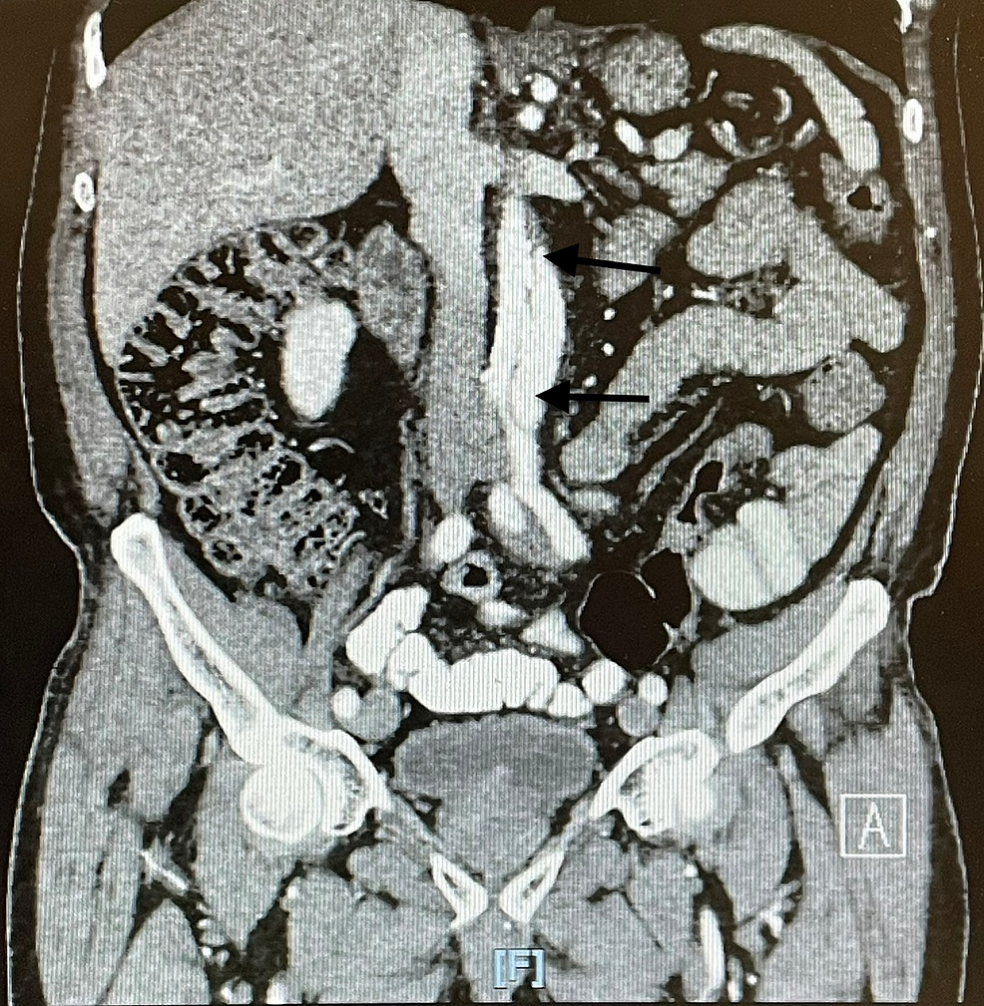
Extensive aortic dissection extending to the iliac arteries

**Figure 5:**
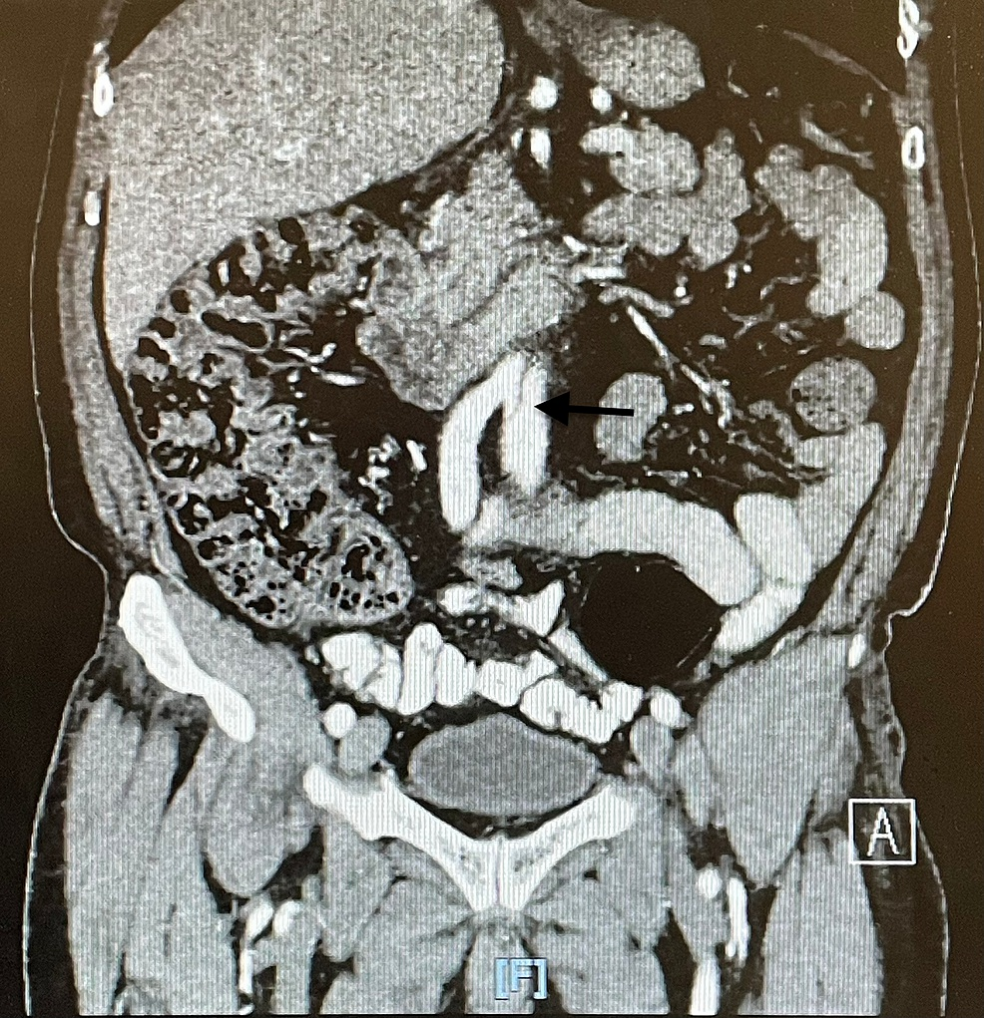
Extensive aortic dissection extending into the iliac arteries

A 64-year-old male, with a significant past medical history of coronavirus disease 2019 (COVID-19) hospitalization status post-intubation and tracheostomy creation, colon cancer status post-chemotherapy treatment in 2018, and retinal vein thrombosis resulting in left eye blindness, was seen in the general surgery clinic for the evaluation of a ventral hernia. The patient was asymptomatic at the time and denied any back or abdominal pain, nausea, or vomiting. For further evaluation, a CT without contrast of the abdomen/pelvis was ordered; however, a CT with contrast was performed instead by mistake. This incidentally revealed an extensive type B aortic dissection extending from the thoracic aorta through the abdominal aorta and to the left iliac artery, as seen in Figures 1-5.

The patient was asymptomatic without any evidence of malperfusion syndrome, but due to the extensive nature of the dissection, he was sent to the emergency department for further evaluation by vascular surgery. An echocardiogram was performed to evaluate the cardiac function, which revealed a normal ejection fraction of 55-60% with no regional wall motion abnormalities. The decision was made by the surgical team to perform a thoracic endovascular aortic repair (TEVAR) given how extensive the dissection was. Stents were placed just distal to the left subclavian artery, proximal to the celiac artery. No complications occurred during or after the procedure. He remained in the hospital for another two days. After a successful procedure and recovery, the patient was discharged home on aspirin 81 mg and atorvastatin 20 mg. The patient followed up at the clinic four weeks later and reported feeling good, and denied any abdominal and/or back pain, nausea, or other symptoms.

## Discussion

Aortic dissection is the most common and devastating manifestation of acute aortic syndrome. Its other manifestations include penetrating aortic ulcer, intramural hematoma, and ruptured thoracic aortic aneurysm [1].

Genetic or acquired diseases predispose individuals to aortic wall weakening, leading to acute aortic syndromes [2]. The International Registry of Acute Aortic Dissection (IRAD) data review showed that aortic dissection is more common in men and presents in them earlier than in women (60 vs. 67 years). Hypertension, atherosclerosis, prior aortic surgeries, and connective tissue disorders are the most common risk factors associated with aortic dissection, the latter being the genetic component, involving the younger population [2-4]. Another review has shown that among people under the age of 40 years with aortic dissection, only 40% had a history of hypertension, and 1% had a history of atherosclerosis [5].

The presentation of aortic dissection varies depending on the extent of involvement. Excruciating pain in the chest, back, or abdomen is the typical presentation associated with aortic dissection. Chest pain is common in ascending dissection (type A) while back and abdominal pain are more commonly associated with descending aortic dissections (type B) [2,6-7]. Although uncommon, painless presentations of aortic dissection have also been reported as in the case discussed above. Patients with painless presentation usually have a history of diabetes, aortic aneurysm, or cardiac surgery [8]. Physical findings associated with the dissection may include blood pressure discrepancies, pulse deficit, and heart murmurs. Focal neurological deficits may also be appreciated with an extensive dissection propagating to the branch arteries.

The diagnosis of acute aortic dissection requires a high index of clinical suspicion based on the presenting symptoms and is an important one to make as a missed diagnosis could lead to a high mortality risk [9-11]. The clinical triad of sharp tearing pain, blood pressure discrepancy (>20-mmHg difference between the right and left arms), and mediastinal widening on chest radiograph could characterize 96% of cases, but cardiovascular imaging remains the ultimate method of diagnosis [12]. CT angiography, MR angiography, and transesophageal echocardiography are the imaging modalities used to diagnose aortic dissection. One may be preferred over the other depending on the availability and patient characteristics. Due to its relatively wider availability, CT angiography is usually preferred as the initial modality of choice [13]. The creation of a false lumen and intimal flap separating it from the true lumen suggests dissection of the aorta. Transesophageal echocardiography is preferred for hemodynamically unstable patients since it has the advantage of bedside availability and being a portable procedure [13]. Digital subtraction aortography is usually reserved for cases where the suspicion of aortic dissection is very high and the noninvasive modalities are inconclusive.

The management of type A aortic dissection involves emergent open-heart surgery. The timely transfer to the operating room should not be interrupted by administering anti-impulse and antihypertensive therapy. The surgical approach involves the excision of the intimal tear and graft replacement of the dissected aorta with or without aortic valve repair. Although limited, endovascular stent grafting is an alternative to surgery for ischemic complications. Treatment of type B aortic dissection depends on the severity of the disease [13-14]. Uncomplicated type B dissection can be successfully managed with antihypertensive and anti-pulsatile therapy. Endovascular or surgical intervention is usually reserved for complicated dissections that involve the progression of the dissection leading to end-organ ischemia, hematoma formation, or frank rupture of the aorta. These complications are associated with a higher mortality rate of 60% [14]. A similar yet different case has been reported in the literature, and it involves an asymptomatic dissection that required immediate action; the patient was a 39-year-old pregnant woman at 35 weeks' gestation who was found to have an aortic dissection on echocardiogram. She had to deliver immediately; however, she refused aortic surgery and never followed up after discharge [15]. Our case involved an incidental finding of an extensive aortic dissection.

## Conclusions

Although type B aortic dissections are not as severe or acute as the type A variant, they require immediate attention as they can evolve into an emergency. Patients are often asymptomatic but can also present acutely, and in such cases, the imaging modality of choice is a CT angiogram in order to visualize the culprit lesion. In our case, the patient was supposed to undergo a CT without contrast for the evaluation of an abdominal hernia but erroneously received one with contrast, which led to the discovery of an extensive aortic dissection extending from the thoracic aorta to the iliac arteries. This report presents an extreme case of an asymptomatic incidental finding of a type B aortic dissection where the patient required emergent surgical repair.

## References

[1] Vilacosta I, San Román JA (2001). Acute aortic syndrome. Heart.

[2] Larson EW, Edwards WD (1984). Risk factors for aortic dissection: a necropsy study of 161 cases. Am J Cardiol.

[3] Hagan PG, Nienaber CA, Isselbacher EM (2000). The International Registry of Acute Aortic Dissection (IRAD): new insights into an old disease. JAMA.

[4] Spittell PC, Spittell JA Jr, Joyce JW, Tajik AJ, Edwards WD, Schaff HV, Stanson AW (1993). Clinical features and differential diagnosis of aortic dissection: experience with 236 cases (1980 through 1990). Mayo Clin Proc.

[5] Januzzi JL, Isselbacher EM, Fattori R (2004). Characterizing the young patient with aortic dissection: results from the International Registry of Aortic Dissection (IRAD). J Am Coll Cardiol.

[6] Pape LA, Awais M, Woznicki EM (2015). Presentation, diagnosis, and outcomes of acute aortic dissection: 17-year trends from the International Registry of Acute Aortic Dissection. J Am Coll Cardiol.

[7] Evangelista A, Isselbacher EM, Bossone E (2018). Insights from the International Registry of Acute Aortic Dissection: a 20-year experience of collaborative clinical research. Circulation.

[8] Park SW, Hutchison S, Mehta RH (2004). Association of painless acute aortic dissection with increased mortality. Mayo Clin Proc.

[9] Huynh N, Thordsen S, Thomas T (2019). Clinical and pathologic findings of aortic dissection at autopsy: review of 336 cases over nearly 6 decades. Am Heart J.

[10] Hansen MS, Nogareda GJ, Hutchison SJ (2007). Frequency of and inappropriate treatment of misdiagnosis of acute aortic dissection. Am J Cardiol.

[11] Chua M, Ibrahim I, Neo X, Sorokin V, Shen L, Ooi SB (2012). Acute aortic dissection in the ED: risk factors and predictors for missed diagnosis. Am J Emerg Med.

[12] von Kodolitsch Y, Schwartz AG, Nienaber CA (2000). Clinical prediction of acute aortic dissection. Arch Intern Med.

[13] Kienzl D, Prosch H, Töpker M, Herold C (2012). Imaging of non-cardiac, non-traumatic causes of acute chest pain. Eur J Radiol.

[14] Nienaber CA, von Kodolitsch Y, Nicolas V (1993). The diagnosis of thoracic aortic dissection by noninvasive imaging procedures. N Engl J Med.

[15] Abo-Salem E, López-Candales A (2014). Diagnosis of asymptomatic aortic dissection during pregnancy using contrast echocardiography. J Cardiol Cases.

